# Three-dimensional printing of mitral valve models using echocardiographic data improves the knowledge of cardiology fellow physicians in training

**DOI:** 10.3389/fcvm.2023.1307994

**Published:** 2023-12-06

**Authors:** Ziad Bulbul, Issam El Rassi, Ramsey Hamade, Hani Tamim, Fadi Bitar

**Affiliations:** ^1^ Department of Pediatrics and Adolescent Medicine, American University of Beirut, Beirut, Lebanon; ^2^ Pediatric Cardiac Surgery, Al Jalila Hospital, Dubai, United Arab Emirates; ^3^ Department of Mechanical Engineering, American University of Beirut, Beirut, Lebanon; ^4^ Department of Biostatistics, American University of Beirut, Beirut, Lebanon

**Keywords:** echocardiography, three-dimensional, mitral valve, printing, educational, cardiology trainees

## Abstract

**Background:**

High fidelity three-dimensional Mitral valve models (3D MVM) printed from echocardiography are currently being used in preparation for surgical repair.

**Aim:**

We hypothesize that printed 3DMVM could have relevance to cardiologists in training by improving their understanding of normal anatomy and pathology.

**Methods:**

Sixteen fellow physicians in pediatric and adult cardiology training were recruited. 3D echocardiography (3DE) video clips of six mitral valves (one normal and five pathological) were displayed and the fellows were asked to name the prolapsing segments in each. Following that, three still images of 3D MVMs in different projections: enface, profile and tilted corresponding to the same MVs seen in the clip were presented on a screen. Participating physicians were presented with a comprehensive questionnaire aimed at assessing whether the 3D MVM has improved their understanding of valvular anatomy. Finally, a printed 3D MVM of each of the valves was handed out, and the same questionnaire was re-administered to identify any further improvement in the participants' perception of the anatomy.

**Results:**

The correct diagnosis using the echocardiography video clip of the Mitral valve was attained by 45% of the study participants. Both pediatric and adult trainees, regardless of the year of training demonstrated improved understanding of the anatomy of MV after observing the corresponding model image. Significant improvement in their understanding was noted after participants had seen and physically examined the printed model.

**Conclusion:**

Printed 3D MVM has a beneficial impact on the cardiology trainees' understanding of MV anatomy and pathology compared to 3DE images.

## 1. Introduction

Since its initial introduction in the 1980s, Three-dimensional (3D) printing of radiographic based imaging has gained momentum as a clinical tool in the medical field over the last few years. From an initial use for industrial work (1), 3D printing first made its debut in the field of clinical care in the 1990s with the manufacture of the first cranial bone model (2). Its use has become widespread in various medical fields from bio-printing tissue organs (3) extending currently to patient-specific implants, prostheses, and replicated anatomical models for education and surgical planning (4, 5). Impressive advancements in the field of Cardiovascular 3D printing have been scored over the last few years and its application is continuously expanding; three-dimensional models of hearts with several congenital defects are currently available as teaching tools (2, 6, 7). Recently, applications of 3D printed modeling of the heart or vascular structures have include interventional preoperative planning and simulation (8–10), patient specific hemodynamic evaluations and testing of novel procedures (11, 12).

**Figure 1:**
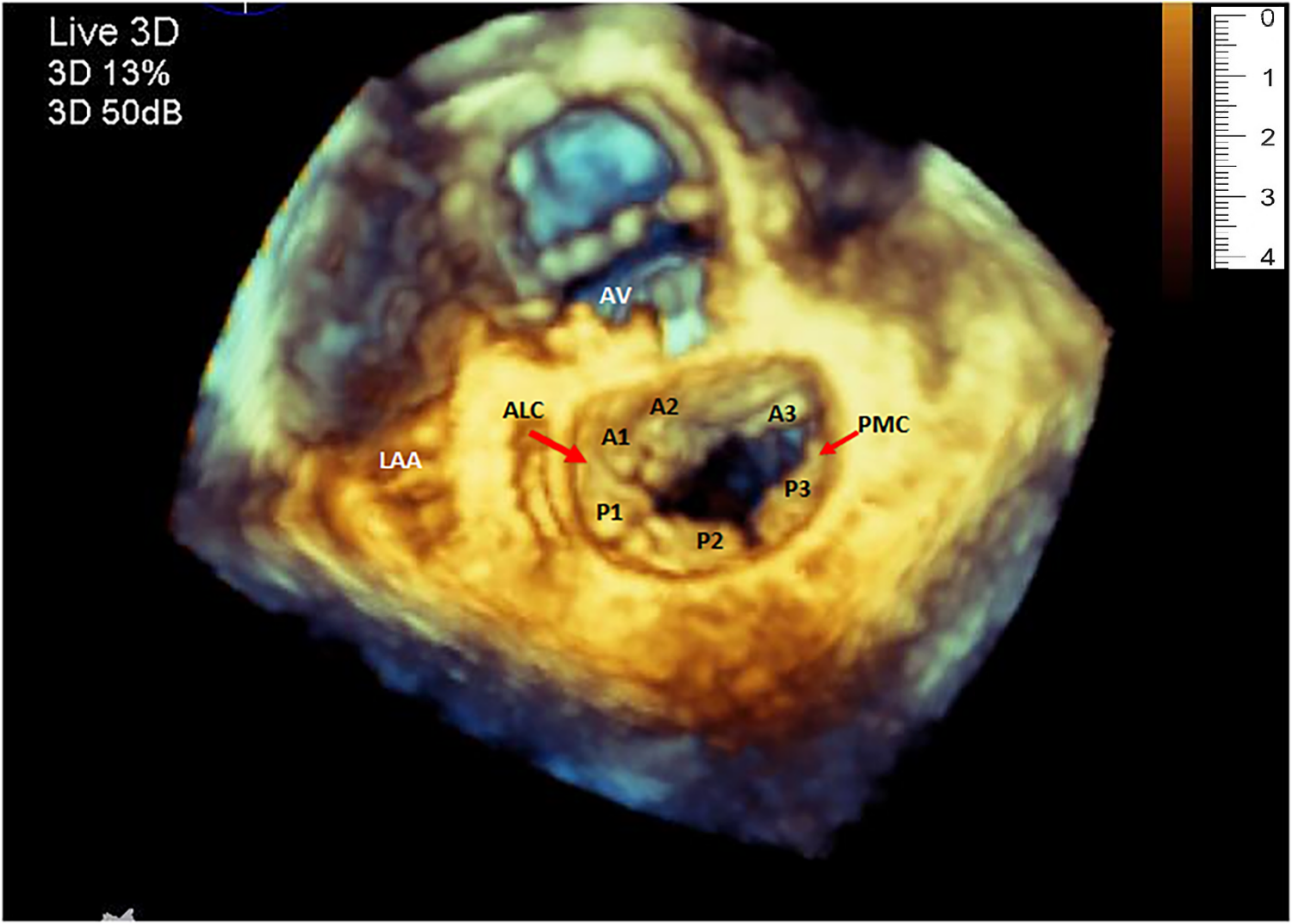
Three-dimensional echocardiography image of the mitral valve showing the normal shape of the annulus, both leaflets and the corresponding segments. (LAA, left atrial appendage; ALC, antero-lateral commissure; PMC, postero-medial commissure).

Although the clinical use of 3D Echocardiography has been around for more than two decades, its value in generating 3D models of cardiac structures has not been thoroughly investigated. Echocardiographic 3D data sets of a mitral valve (Figure 1), obtained via the transesophageal window, have been shown to have enough spatial and temporal resolution for clinical use. Successful attempts to 3D print from transesophageal echo for the evaluation of and closure of paravalvular leaks of the mitral valves have been reported (13, 14).

Currently there are several commercial software packages, that have transformed and simplified image data segmentation and 3D rendering. As important, was the unlocking of vendor-specific tags by establishing an open-source workflow for generating 3D anatomic models from routine clinical echocardiographic data sets (15, 16).

**Figure 2:**
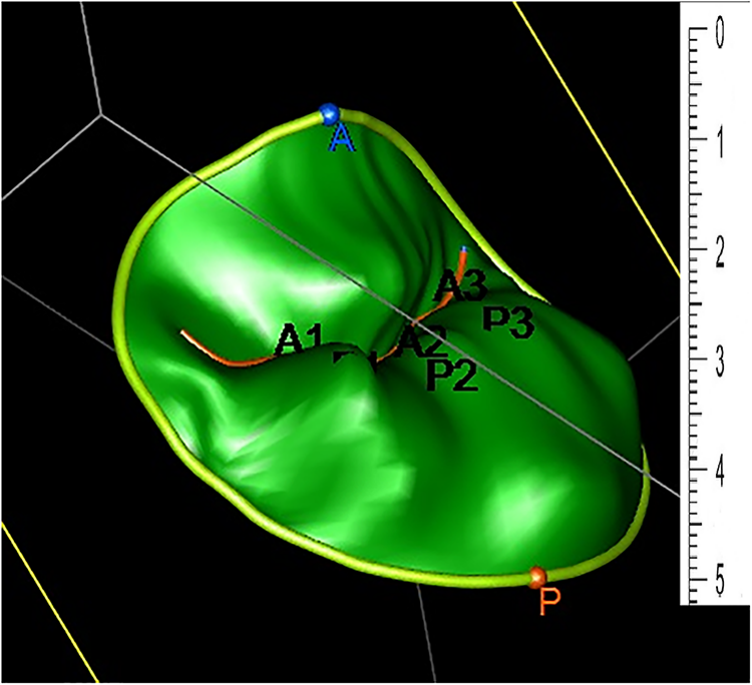
Three-dimensional geometrical Model generated based on an echocardiographic image. The two leaflets are divided into a total of six scallops: A1, A2, A3 (anterior) and P1, P2, P3 (posterior).

The rendered dynamic 3DE based images of the MV are an excellent adjunct to the conventional two-dimensional echocardiography for surgical repair. However, projecting a 3D structure on a flat screen inevitably affects the viewer's depth perception. In addition to the rendered 3D echocardiographic image, a 3D geometrical Model (Figure 2) can be generated based on an echocardiographic image. The model can be used to perform several measurements that are crucial for the understanding of the structures of the MV and the planning of surgical intervention. Unlike 3DE images, the same MVM when printed in 3D, provides excellent depth while conserving details.

**Figure 3:**
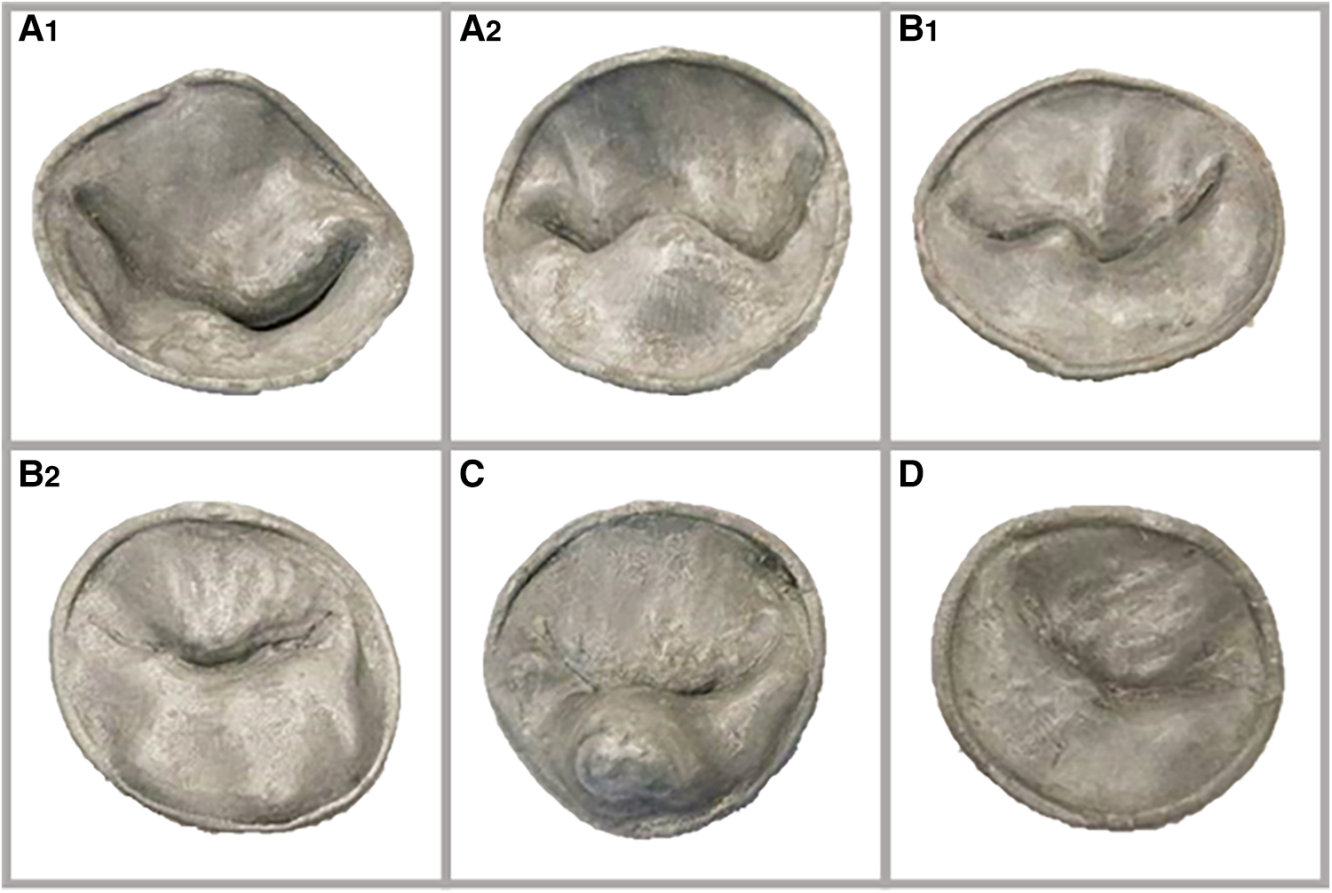
(**A1**) and (**A2**): two valves with single leaflet segment prolapse; (**B1**) and (**B2**): two valves with at least two segments prolapse involving both leaflets, with lack of leaflet coaptation in one. (**C**): valve with a flail segment. (**D**): normal valve.

We hypothesize that a printed 3D models of MV (Figure 3) could objectively improve the understanding of the MV geometry and depth perception, for cardiologists in-training, further enhancing their understanding of normal valve anatomy and pathology.

## 2. Methods

This study is a cross sectional questionnaire-based educational study administered to cardiology physicians in training, between June 2017 and March 2018, at the American University of Beirut-Medical Center (AUBMC). The study was approved by the research Institutional Review Board of AUBMC. Cardiology fellows consented to be part of the study, and emphasis was placed on the fact that participation is voluntary and that they could withdraw from the study at any point in time if they chose to do so. Moreover, all data collected including videos, clinical models, and fellows’ details (except for their year of training and subspecialty) were anonymous. Confidentiality was maintained throughout the study. Sixteen cardiology fellows at different levels of training (eleven adult cardiology and five pediatric cardiology) were recruited and completed the study.

Imaging of the MV and the acquisition of 3D data sets were completed using a (t7-1) transesophageal probe and (iE-33) echocardiography platforms (Philips, Eindhoven Netherlands). Rendered 3D images of the mitral valve were generated using QLAB software v.8 (Philips, Eindhoven Netherlands) and were saved as anonymous video clips, (Audio Video Interleave format, Microsoft, Washington, USA).

Native 3D data sets were imported into commercially available software (Image Arena, TomTec GmbH. Munich, Germany), where another, plug in semi-automated specialized software (MVA package 2.1) analyzes and tracks the mitral valve and generates a geometrical model of the valve to include the annulus, leaflets and coaptation line. Those models were saved in JPEG (Joint Photographic Experts Group) format and exported as stereolithographic files (stl).

Files of the models were printed using polylactic acid (PLA), a biodegradable thermoplastic aliphatic polyester with a tensile strength of 49.5 MPa and Shore hardness of 83D, on a commercial Ultimaker 2 + R 3D printer, with range of resolutions from 20 microns all the way to a sizeable 600 microns (Ultimaker B.V. Watermolenweg, Netherlands). The fidelity of the resulting hard models was highly conserved, as they resembled the original echocardiographic model pictures.

A total of six (One normal and five pathological) valves were rendered in 3D and saved as video clips. For each 3D rendered MV, a 3D model was generated, saved as still image, and printed in 3D. Pathological models included: two valves with single leaflet segment prolapse (Figures 3A1,A2), two valves with at least two segments prolapse involving both leaflets and lack of leaflet coaptation (Figures 3B1,B2), and a fifth valve, marked as valve C, demonstrated a flail segment. Valve D was the normal valve (Figure 3).

**Figure 4:**
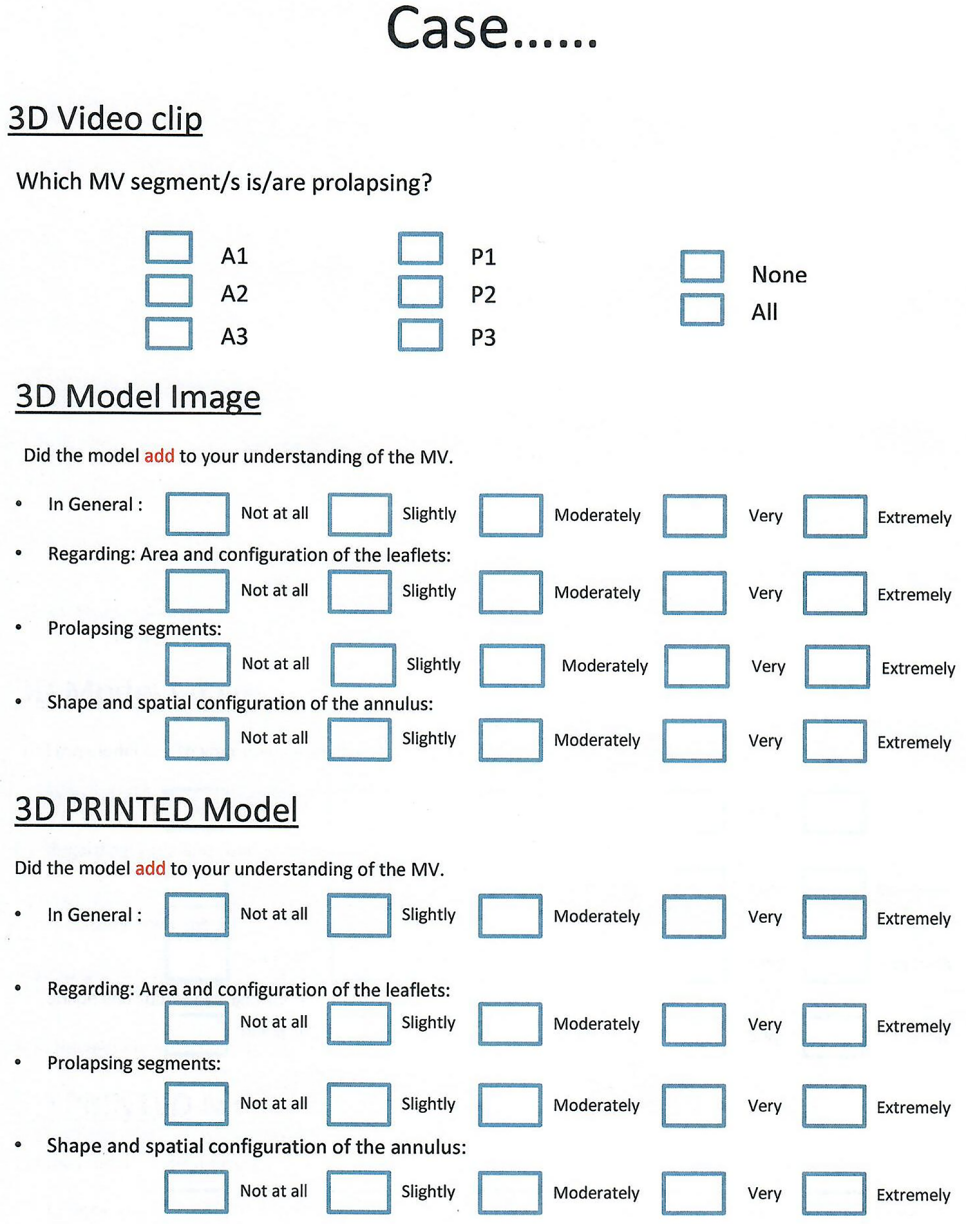
Questionnaire designed to elicit whether the 3D printed model improved participant perception and knowledge about the mitral valve.

To test our hypothesis, a PowerPoint (Microsoft incorporation- Washington, USA) based questionnaire was prepared. The questionnaire was specifically designed for this project to elicit whether the 3D printed model improved participant perception and knowledge about the MV (Figure 4).

Movie clips of 3D echo of the 6 MVs were initially projected to the study participants, after which they were asked to name the pathological segment(s) of the MV leaflets, if any existed. Following that, pictures of the 3D generated models of the same valves were sequentially projected on a flat screen. Participants were asked if that had added to their understanding of the valve regarding the following categories: (1) anatomy in general, (2) area and prolapse of the leaflets, and (3) shape of the annulus. Finally, the corresponding 3D printed MV models were handed over to the participants for tangible assessment. The same questionnaire was administered once again following their assessment of the printed model.

### 2.1. Statistical analysis

Data were entered and analyzed using the Statistical Package for Social Sciences program (SPSS Inc, Chicago, IL). For each case, the number and percentage of fellows giving the correct answer and whether it improved their understanding of MV anatomy and pathology was calculated and presented as a mean and standard deviation. Each fellow was asked to assign a response to each of the four questions describing how a certain image modality (3D model or 3D printed respectively) improved their understanding of MV anatomy and pathology. The available answers were: “extremely”, “very”, “moderately”, “slightly”, and “not at all”. For the presentation of the perception data, we have created three groups of responses. A negative perception as indicated by “not at all” and “slightly” answers, a neutral perception as indicated by “moderately” answer, and a positive perception as indicated by “very” and “extremely”. Percentages of negative, neutral, and positive responses were calculated for each category in all 6 cases.

In order to assess the overall responses and compare outcomes between adult and pediatric fellows on one hand, and between junior (first-year trainees) and senior fellows (second- and third-year trainees) on the other; number and percentages of positive answers were compared and the difference in mean scores was calculated using the Mann–Whitney test, where *p*-value <0.05 was used to indicate statistical significance.

## 3. Results

### 3.1. Video clips

Based on the video clips of the valves (Table 1), fourteen participants (87.5%) identified the normal case correctly. Twelve (75%) and seven (44%) of them named the correct prolapsing segment in the two models with one pathological segment. However, six (37.5%), four (25%) and only one (6.3%) participant identified correctly, all the pathological segments in the last three complex cases (with more than one prolapsing segment present).

**Table 1:** Number and percentages of the participants who made the correct diagnosis of pathological segment of mitral valve based on video clip.

Video of 3D MV^a^	Case 1	Case 2	Case 3	Case 4	Case 5	Case 6
N^b^ (%)	14 (87.5%)	7 (43.8%)	12 (75.0%)	6 (37.5%)	4 (25.0%)	1 (6.3%)

^a^ MV, mitral valve.

^b^ N, number of participants with correct answer.

Total number of participants =16.

### 3.2. Picture of the 3D model

Following the projection of the picture of the 3D constructed model of the valve, (44.8%), (41.7%), (40.6%) and (51.6%) of the group agreed that the picture of the model improved their understanding of the general appearance of the MV, area of the valve, prolapsing segments, and shape of the annulus respectively.

Among the junior fellows: (38.1%) indicated that the picture of the 3D model improved their general understanding of the mitral valve anatomy; (45.2%) stated that it improved their perception of the valve's area, (46.3%) agreed it changed positively their understanding of the leaflet prolapse, and 40.5% indicated that it improved their understanding of the shape of the annulus. This is in comparison to the senior fellows of whom (42.6), (44.4), (55.6) and (42.6)% agreed that the picture of 3D model improved their understanding of the valve in general, it's area, prolapsing leaflets, and the shape of the annulus respectively (Table 2).

**Table 2:** The change in fellows’ perception of mitral valve anatomy after examination of 3D printed model as compared to the picture of 3D model.

All Trainees (*n* = 16)	Response	Picture of 3D model	Print of 3D model	*p*-value
		(%)	(%)	
General appearance	Negative	(24.0)	(8.3)	
	Neutral	(35.4)	(15.6)	
	Positive	(40.6)	(76.0)	<0.0001
Area of valve	Negative	(25.0)	(9.5)	
	Neutral	(30.2)	(15.8)	
	Positive	(44.8)	(74.7)	<0.0001
Prolapsing segments	Negative	(25.3)	(5.3)	
	Neutral	(23.2)	(15.8)	
	Positive	(51.6)	(78.9)	<0.0001
Shape of annulus	Negative	(22.9)	(9.4)	
	Neutral	(35.4)	(21.9)	
	Positive	(41.7)	(68.8)	<0.0001
Junior trainees (*n* = 7)				
General appearance	Negative	(19.0)	(2.4)	
	Neutral	(42.9)	(23.8)	
	Positive	(38.1)	(73.8)	<0.0001
Area of valve	Negative	(19.0)	(2.4)	
	Neutral	(35.7)	(22.0)	
	Positive	(45.2)	(75.6)	= 0.001
Prolapsing segments	Negative	(19.5)	(4.9)	
	Neutral	(34.1)	(19.5)	
	Positive	(46.3)	(75.6)	= 0.001
Shape of annulus	Negative	(16.7)	(4.8)	
	Neutral	(42.9)	(35.7)	
	Positive	(40.5)	(59.5)	= 0.02
Senior trainees (*n* = 9)				
General appearance	Negative	(27.8)	(13.0)	
	Neutral	(29.6)	(9.3)	
	Positive	(42.6)	(77.8)	<0.0001
Area of valve	Negative	(29.6)	(14.8)	
	Neutral	(25.9)	(11.1)	
	Positive	(44.4)	(74.1)	<0.0001
Prolapsing segments	Negative	(29.6)	(5.6)	
	Neutral	(14.8)	(13.0)	
	Positive	(55.6)	(81.5)	<0.0001
Shape of annulus	Negative	(27.8)	(13.0)	
	Neutral	(29.6)	(11.1)	
	Positive	(42.6)	(75.9)	<0.0001
Adult trainees (*n* = 11)				
General impression	Negative	(6.7)	(0.0)	
	Neutral	(26.7)	(23.3)	
	Positive	(66.7)	(76.7)	= 0.10
Area of valve	Negative	(6.7)	(0.0)	
	Neutral	(23.3)	(20.7)	
	Positive	(70.0)	(79.3)	= 0.06
Prolapsing segments	Negative	(10.3)	(3.4)	
	Neutral	(20.7)	(17.2)	
	Positive	(69.0)	(79.3)	= 0.03
Shape of annulus	Negative	(6.7)	(0.0)	
	Neutral	(26.7)	(26.7)	
	Positive	(66.7)	(73.3)	= 0.10
Pediatric Trainee (*n* = 5)				
General impression	Negative	(31.8)	(12.1)	
	Neutral	(39.4)	(12.1)	
	Positive	(28.8)	(75.8)	<0.0001
Area of valve	Negative	(33.3)	(13.6)	
	Neutral	(33.3)	(13.6)	
	Positive	(33.3)	(72.8)	<0.0001
Prolapsing segments	Negative	(31.8)	(6.1)	
	Neutral	(24.4)	(15.2)	
	Positive	(43.8)	(78.7)	<0.0001
Shape of annulus	Negative	(30.3)	(13.6)	
	Neutral	(39.4)	(19.7)	
	Positive	(30.3)	(66.7)	<0.0001

*P*-value is calculated for the positive response.

### 3.3. Inspection of printed model

Inspection of printed model has improved the fellows understanding of the mitral valve in all 4 categories according to the percentages of the positive responses obtained: (1) general appearance of the valve (76%); (2) area of the valve (74.7%); (3) prolapsing segments (78.9%) and (4) shape of the annulus (68.8%). When comparing fellows according to their level of training; senior fellows were more likely to agree that the 3D printed model was helpful in improving their understanding of the mitral valve in all categories when compared to their junior counterparts: (77.8%) of senior fellows agreed the 3D printed model improved their understanding of the general appearance of the valve vs. (73.8%) for junior fellows (*p* = 0.05). The results were similar regarding the shape of the annulus: (75.9%) vs. (59.5%) (*p* = 0.01) and their understanding of the prolapsing segments: 81.5% vs. 75.6% (*p* = 0.03). However, there was no statistical difference between junior and senior fellows for whether the 3D printed model improved their understanding of the valve's area:75.6% vs. 74.1% (*p* = 0.05) (Table 3).

**Table 3:** Comparing percentages of the fellows’ responses on the improvement in their perception of mitral valve following inspection the 3D model printed model, according to years of training. *P*-value is calculated for the positive response.

Printed model		Level of training		*p*-value
		Junior	Senior	
General appearance	Negative	(2.4%)	(13.0%)	
	Neutral	(23.8%)	(9.3%)	
	Positive	(73.8%)	(77.8%)	= 0.05
Area of valve	Negative	(2.4%)	(14.8%)	
	Neutral	(22.0%)	(11.1%)	
	Positive	(75.6%)	(74.1%)	= 0.73
Prolapsing segments	Negative	(4.9%)	(5.6%)	
	Neutral	(19.5%)	(13.0%)	
	Positive	(75.6%)	(81.5%)	= 0.03
Shape of annulus	Negative	(4.8%)	(13.0%)	
	Neutral	(35.7%)	(11.1%)	
	Positive	(59.5%)	(75.9%)	= 0.01

### 3.4. Picture vs. print of 3D model

When comparing the picture of the 3D model to its corresponding 3D print, the whole group perceived that the printed model had significantly improved their understanding of the mitral valve over the model picture in all four categories: 76% vs. 46% (*p* < 0.0001) for the general appearance, 74.7% vs. 44.8% (*p* < 0.0001) for the area, 78.9% vs. 51.6% for prolapsing segments (*p* < 0.0001) and 69% vs. 42% (*p* < 0.0001) for shape of the annulus (Table 2).

These results were the same regardless of the year of training; Junior fellows agreed the 3D printed model improved their understanding of the mitral valve in all four categories when compared to the picture of the 3D model: 73.8% vs. 38.1% (*p* < 0.0001) for general appearance,75.6% vs. 45.2% for the valve's area (*p* < 0.001), 75.6% vs. 46.3% (*p* < 0.001) for prolapsing segments: 59.5% vs. 40.5% (*p* = 0.02) for the shape of the mitral valve. Senior fellows followed the same trend: 77.8% vs. 42.6% (*p* < 0.0001) for the general appearance, 74.1% vs. 44.4% (*p* < 0.0001) for the area, 81.5% vs. 55.6% (*p* < 0.0001) for prolapsing segments, and 75.9% vs. 42.6% (*p* < 0.0001) for the shape of the annulus.

## 4. Discussion

The complexity of the mitral valve has been challenging to image ever since the initial stages of echocardiography. Advancement in 2DE during the last three decades; however, has revolutionized the cardiologists' understanding of the mitral valve over the initial “B” and “M” echocardiographic imaging modes. The introduction of the mono- and biplane electronic phased-array probes in 1982, on the other hand establishes a new landmark in the clinical use of Trans Esophageal Echocardiograms (TEE) (17). Two-dimensional TEE and its application on the mitral valve imaging (18, 19) has expanded the scope of mitral valve repair surgery (20). Before the introduction of 3D TEE, the combination of perioperative TEE data with the results of direct intraoperative inspection of the MV in an arrested heart was the standard practice surgeons used in making decision regarding mitral valve repair. Despite all advancement at that stage, the understanding of the MV pathology requires the assimilation of two-dimensional images with direct intraoperative valve findings. Ahmed et al. reported that this approach could be challenging for less experienced echocardiographers; especially in cases of complex MV prolapse involving multiple MV segments (21).

Training a cardiologist on echocardiography is a tedious and lengthy process. The biggest challenge for a trainee is to mentally reconstruct a 3D image from multiple slices of 2D planes of a given structure. To achieve such a purpose, the advantage of 3DE over 2D TEE is obvious, especially for a complex structure like the MV. Furthermore, La Canna et al. alluded to an additional diagnostic value of 3D TEE, in that it provides more accurate mapping of MV prolapse and further details of MV anatomy (22).

Currently, 3D images rendered from 3DE are projected on 2D monitors. The mere fact of projecting a three-dimensional structure on a flat screen is a significant shortcoming, despite the use of different hues and shadows to improve depth perception.

Recently, 3D printing has gained momentum in the medical field. Anatomical structures from different systems of the body are printed in a very high degree of accuracy (4, 5). Moreover, the cost of printing has decreased significantly, and most centers nowadays can print their 3D images.

While printing cardiac structures from CT and MRI scans are done routinely nowadays; printing from echocardiographic data has not yet been perfectedt. This is mainly due to the relatively low special resolution of 3DE images. Mahmoud et al. (23) and others have printed 3D models of the mitral valve generated from echocardiographic data. The resulting models closely mimicked the original echocardiographic pictures. This imaging modality however still requires some refinement to better describe leaflet coaptation, chordae, papillary muscles and left ventricular geometry (24).

In the field of surgery, the improvement in MV surgical outcomes coinciding with the use of 3D based images was highlighted by Beraud and his group (25). Hien et al. had shown in a multicenter study that the use of 3DE of the Mitral valve improves the understanding of prolapse segments (26). The fact that this was true for beginners and experienced echocardiographers alike was of interest. Taking it one step further, Hadeed and his group in a brief communication, proposed the use of printed 3D models of complex congenital lesions as a roadmap for surgical repair (27). Recently, Premyodhin et al. published their experience in the use of a specially constructed MV molds, that simulated natural tissue in the preoperative simulation of robotic mitral valve repair (28).

From the educational point of view, Biglino et al, in a recent publication showed the benefit of using 3D models of the heart as an educational tool for the training of both adult and pediatric cardiac nurses (29). Costello et al. found that even pediatric residents' understanding of lesions like ventricular septal defect could be further enhanced by the printed model as compared to educational seminars in anatomy and echocardiography. They proposed that 3D printed heart models can be effectively incorporated into a simulation-based congenital heart disease and critical care training curriculum for pediatric resident physicians (10).

One of the advantages of the physical inspection of the 3D model over a 2D projection of its image could be explained by the lack of the depth perception that is intrinsic to projecting a 3D structure onto a 2D screen.

In this study, we sought to prove that a physical model of the mitral valve, printed based on MV echocardiographic data, can enhance the understanding of the normal and abnormal anatomy of mitral valves even at a high level of training. The Mitral valve models with leaflets and annuli were printed from rigid materials only, for the purpose of replicating systolic static leaflet and annular geometry. While this method serves the purpose of this study; patient-specific functional models of the mitral valve and with deformable leaflets, recently been used to plan mitral valve catheter repair procedures (30) would have been of an added value. Our data; however, clearly shows that the MV model, when physically examined by the cardiology fellows, did significantly improve their understanding even after seeing a 3D image of the same model. This observation was not only valid for the prolapsing segments but also for the area and shape of the valve, and the complex anatomy of the annulus. This was the case irrespective of the level of training of the cardiology fellow.

In the future, further advancement in the spatial and temporal resolution of echo generated 3D images along with the introduction of fused dual-material 3D printing, will permit the printing of actual structures of normal as well as congenitally deformed hearts from echocardiographic data in high fidelity and accuracy. Eventually, combining valves and sub valvular apparatus printed from echocardiographic data with heart models printed from CT or MRI scans will further improve our understanding of congenital heart disease. Such models will directly and positively impact our ability to educate trainees, select patients for structural heart interventions, and potentially revise and improve the use of intra-cardiac devices. Ultimately, incorporating artificial intelligence in the application of 3D printing, and computational modeling, will modify patient care and the way physicians are trained (31).

## 5. Conclusion

Three-dimensional models of MV manufactured using echocardiographic data and printed with 3D technology positively impact the understanding of both adult and pediatric cardiology trainees regarding the MV anatomy and pathology. It might further enhance the diagnostic power of 3DE especially in complex cases of MV disease.

### 5.1. Study limitations

Although the number of study participants was small, the results were coherent for the superiority of the 3D printed model. This study could thus serve as a starting point for larger studies to assess the effectiveness of 3D models integration in medical education. Larger studies utilizing more participants and printed models using integrated flexible and rigid printing materials are needed.

## Data Availability

The raw data supporting the conclusions of this article will be made available by the authors, without undue reservation.

## References

[1] Sun Z Squelch A. 3D Printed models of complex anatomy in cardiovascular disease. Heart Res Open J. (2015) 2(3):103–8. 10.17140/HROJ-2-118

[2] Kim MS Hansgen AR Wink O Quaife RA Carroll JD. Rapid prototyping: a new tool in understanding and treating structural heart disease. Circulation. (2008) 117(18):2388–94. 10.1161/CIRCULATIONAHA.107.740977 18458180

[3] Daemen JH Heuts S Olsthoorn JR Maessen JG Sardari Nia P. Mitral valve modelling and three-dimensional printing for planning and simulation of mitral valve repair. Eur J Cardiothorac Surg. (2019) 55(3):543–51. 10.1093/ejcts/ezy306 30202862

[4] Frame M Leach W. DIY 3D Printing of custom orthopaedic implants: a proof of concept study. Surg Technol Int. (2014) 24:314–7. 24574013

[5] Gerstle TL Ibrahim AM Kim PS Lee BT Lin SJ. A plastic surgery application in evolution: three-dimensional printing. Plast Reconstr Surg. (2014) 133(2):446–51. 10.1097/01.prs.0000436844.92623.d3 24469175

[6] Garekar S Bharati A Chokhandre M Mali S Trivedi B Changela VP Clinical application and multidisciplinary assessment of three dimensional printing in double outlet right ventricle with remote ventricular septal defect. World J Pediatr Congenit Heart Surg. (2016) 7(3):344–50. 10.1177/2150135116645604 27142402

[7] Olivieri LJ Krieger A Loke YH Nath DS Kim PC Sable CA. Three-dimensional printing of intracardiac defects from three-dimensional echocardiographic images: feasibility and relative accuracy. J Am Soc Echocardiogr. (2015) 28(4):392–7. 10.1016/j.echo.2014.12.016 25660668

[8] Schmauss D Haeberle S Hagl C Sodian R. Three-dimensional printing in cardiac surgery and interventional cardiology: a single-centre experience. Eur J Cardiothorac Surg. (2015) 47(6):1044–52. 10.1093/ejcts/ezu310 25161184

[9] Chaowu Y Hua L Xin S. Three-dimensional printing as an aid in transcatheter closure of secundum atrial septal defect with rim deficiency: in vitro trial occlusion based on a personalized heart model. Circulation. (2016) 133(17):e608–10. 10.1161/CIRCULATIONAHA.115.020735 27143157

[10] Costello JP Olivieri LJ Su L Krieger A Alfares F Thabit O Incorporating three-dimensional printing into a simulation-based congenital heart disease and critical care training curriculum for resident physicians. Congenit Heart Dis. (2015) 10(2):185–90. 10.1111/chd.12238 25385353

[11] Yang DH Kang JW Kim N Song JK Lee JW Lim TH. Myocardial 3-dimensional printing for septal myectomy guidance in a patient with obstructive hypertrophic cardiomyopathy. Circulation. (2015) 132(4):300–1. 10.1161/CIRCULATIONAHA.115.015842 26216088

[12] Kheradvar A Groves EM Simmons CA Griffith B Alavi SH Tranquillo R Emerging trends in heart valve engineering: part III. Novel technologies for mitral valve repair and replacement. Ann Biomed Eng. (2015) 43(4):858–70. 10.1007/s10439-014-1129-y 25287646

[13] Jedrzejek M Kozlowski M Peszek-Przybyla E Jadczyk T Pysz P Wojakowski W Mitral paravalvular leak 3D printing from 3D-transesophageal echocardiography. Anatol J Cardiol. (2023) 27(10):573–9. 10.14744/AnatolJCardiol.2023.3008 37288866 PMC10541781

[14] Jędrzejek M Peszek-Przybyła E Jadczyk T Zemik J Piprek P Pysz P 3D Printing from transesophageal echocardiography for planning mitral paravalvular leak closure—feasibility study. Postepy Kardiol Interwencyjnej. (2023) 19(3):270–6. 10.5114/aic.2023.131481 37854960 PMC10580856

[15] Hosny A Shen T Kuo AS Long D Andrawes MN Dilley JD. Unlocking vendor-specific tags: three-dimensional printing of echocardiographic data sets. J Thorac Cardiovasc Surg. (2018) 155(1):143–145.e1. 28942976 10.1016/j.jtcvs.2017.08.064

[16] Byrne N Velasco Forte M Tandon A Valverde I Hussain T. A systematic review of image segmentation methodology, used in the additive manufacture of patient-specific 3D printed models of the cardiovascular system. JRSM Cardiovasc Dis. (2016) 5:2048004016645467. 10.1177/2048004016645467 27170842 PMC4853939

[17] Souquet J Hanrath P Zitelli L Kremer P Langenstein B Schluter M. Transesophageal phased array for imaging the heart. IEEE Trans Biomed Eng. (1982) 29(10):707–12. 10.1109/TBME.1982.324864 7173935

[18] Lambert A-S Miller JP Merrick SH Schiller NB Foster E Muhiudeen-Russell I Improved evaluation of the location and mechanism of mitral valve regurgitation with a systematic transesophageal echocardiography examination. Anesth Analg. (1999) 88(6):1205–12. 10.1097/00000539-199906000-00004 10357320

[19] Shanewise JS Cheung AT Aronson S Stewart WJ Weiss RL Mark JB ASE/SCA guidelines for performing a comprehensive intraoperative multiplane transesophageal echocardiography examination: recommendations of the American Society of Echocardiography council for intraoperative echocardiography and the society of cardiovascular anesthesiologists task force for certification in perioperative transesophageal echocardiography. J Am Soc Echocardiogr. (1999) 12(10):884–900. 10.1016/S0894-7317(99)70199-9 10511663

[20] Sidebotham DA Allen SJ Gerber IL Fayers T. Intraoperative transesophageal echocardiography for surgical repair of mitral regurgitation. J Am Soc Echocardiogr. (2014) 27(4):345–66. 10.1016/j.echo.2014.01.005 24534653

[21] Ahmed S Nanda NC Miller AP Nekkanti R Yousif AM Pacifico AD Usefulness of transesophageal three-dimensional echocardiography in the identification of individual segment/scallop prolapse of the mitral valve. Echocardiography. (2003) 20(2):203–9. 10.1046/j.1540-8175.2003.03010.x 12848691

[22] La Canna G Arendar I Maisano F Monaco F Collu E Benussi S Real-time three-dimensional transesophageal echocardiography for assessment of mitral valve functional anatomy in patients with prolapse-related regurgitation. Am J Cardiol. (2011) 107(9):1365–74. 10.1016/j.amjcard.2010.12.048 21371680

[23] Mahmood F Owais K Taylor C Montealegre-Gallegos M Manning W Matyal R Three-dimensional printing of mitral valve using echocardiographic data. JACC Cardiovasc Imaging. (2015) 8(2):227–9. 10.1016/j.jcmg.2014.06.020 25457770

[24] Witschey WR Pouch AM McGarvey JR Ikeuchi K Contijoch F Levack MM Three-dimensional ultrasound-derived physical mitral valve modeling. Ann Thorac Surg. (2014) 98(2):691–69. 10.1016/j.athoracsur.2014.04.094 25087790 PMC4382862

[25] Beraud A-S Schnittger I Miller DC Liang DH. Multiplanar reconstruction of three-dimensional transthoracic echocardiography improves the presurgical assessment of mitral prolapse. J Am Soc Echocardiogr. (2009) 22(8):907–13. 10.1016/j.echo.2009.05.007 19553082

[26] Hien MD Großgasteiger M Rauch H Weymann A Bekeredjian R Rosendal C. Experts and beginners benefit from three-dimensional echocardiography: a multicenter study on the assessment of mitral valve prolapse. J Am Soc Echocardiogr. (2013) 26(8):828–34. 10.1016/j.echo.2013.04.015 23706343

[27] Hadeed K Dulac Y Acar P. Three-dimensional printing of a complex CHD to plan surgical repair. Cardiol Young. (2016) 26(7):1432–4. 10.1017/S1047951116000755 27321706

[28] Premyodhin N Mandair D Ferng AS Leach TS Palsma RP Albanna MZ 3D Printed mitral valve models: affordable simulation for robotic mitral valve repair. Interact Cardiovasc Thorac Surg. (2017) 26(1):71–6. 10.1093/icvts/ivx243 PMC7868959 29049538

[29] Biglino G Capelli C Koniordou D Robertshaw D Leaver LK Schievano S Use of 3D models of congenital heart disease as an education tool for cardiac nurses. Congenit Heart Dis. (2017) 12(1):113–8. 10.1111/chd.12414 27666734

[30] Little SH Vukicevic M Avenatti E Ramchandani M Barker CM. 3D Printed modeling for patient-specific mitral valve intervention: repair with a clip and a plug. JACC Cardiovasc Interv. (2016) 9(9):973–5. 10.1016/j.jcin.2016.02.027 27151611

[31] Wang DD Qian Z Vukicevic M Engelhardt S Kheradvar A Zhang C 3D Printing, computational modeling, and artificial intelligence for structural heart disease. JACC Cardiovasc Imaging. (2021) 14(1):41–60. 10.1016/j.jcmg.2019.12.022 32861647

